# Association between preterm delivery and bacterial vaginosis with or without treatment

**DOI:** 10.1038/s41598-018-36964-2

**Published:** 2019-01-24

**Authors:** Masao Shimaoka, Yoshie Yo, Kunihiko Doh, Yasushi Kotani, Ayako Suzuki, Isao Tsuji, Masaki Mandai, Noriomi Matsumura

**Affiliations:** 1 Department of Obstetrics and Gynecology, Kindai University Faculty of Medicine, Osakasayama, Japan; 2 Department of Obstetrics and Gynecology, PL Hospital, Tondabayashi, Japan; 3 Department of Gynecology and Obstetrics, Kyoto University, Kyoto, Japan

## Abstract

The relationship between bacterial vaginosis (BV) and preterm delivery has become well known in recent years, although there are few studies on: (i) the differences in test results during the early gestational (EGP) and middle gestational (MGP) periods; (ii) the significance of the intermediate (I) group that does not develop overt BV; or (iii) the therapeutic effects of metronidazole. We performed a retrospective study to analyze the relationship between the vaginal bacterial status and the preterm delivery rate. Without treatment, the preterm delivery rate was higher in the BV subgroup than in the I and normal (N) subgroups (p = 0.021) in the EGP, whereas the rates in the BV and I subgroups were higher than in the N subgroup in the MGP (p = 0.0003). Although treatment of BV by metronidazole vaginal tablets significantly increased the N subgroup in the MGP (p = 0.020), there was no significant improvement in the preterm delivery rate. Decreasing the rate of preterm delivery requires development of treatment methods that will further increase the percentage of patients who test N during the MGP after BV during the EGP.

## Introduction

Bacteria such as *Lactobacillus lactis* have an intravaginal cleansing effect in normal pregnancies, minimizing the presence of common bacterial species^1^. Recent high-throughput sequencing of 16 S rRNA gene of the vaginal bacterial communities of pregnant women showed that vaginal microbiome becomes more stable and less diverse as pregnancy progresses, which confers a protective role against ascending infection of the genital tract^2^. However, there are intravaginal microorganisms other than *Lactobacillus* species in some pregnancies that cause chorioamnionitis. This is because the normal flora (commensal bacteria) that colonize the vagina during pregnancy do not cause inflammatory conditions or vaginitis, such as occurs with infection by pathogenic bacteria. An imbalance in the normal vaginal bacteria is therefore known as bacterial vaginosis (BV). Chorioamnionitis is the main cause of preterm delivery, and various previous reports have stated that chorioamnionitis occurs against a background of BV^3^. When BV causes vaginitis or cervicitis and then progresses to inflammation of all fetal membranes, it can in turn cause premature rupture of membranes and labor^4,5^. It is therefore important to manage BV first in order to prevent preterm delivery. Metronidazole is the most effective antibiotic for BV. A 2007 Cochrane review stated that treating BV with antibiotics up to gestational week (GW) 20 prevents preterm delivery^6^. However, many clinical trials and a 2013 Cochrane review reported no preventive effects on preterm delivery, despite administration of treatment for BV^7–9^. This discrepancy mandates further research on the role of BV treatment in this setting.

We performed BV screening on all pregnant women during the early gestational period (EGP) and middle gestational period (MGP) between 2004 and 2008 at our hospital. These patients remained untreated for BV. We also performed BV screening on all pregnant women during the EGP from 2010 onwards at our hospital. All of the latter patients diagnosed with BV were treated with metronidazole vaginal tablets. To begin with, this study retrospectively investigates the natural course of BV and its association with preterm delivery. We then analyze the therapeutic effects of metronidazole vaginal tablet administration and its preventive effects on preterm delivery.

## Materials and Methods

### Patients

The current study was retrospective. All the study protocols were approved by the Kindai University Faculty of Medicine Ethics Committee. Informed consent was obtained from all the patients. All the methods were carried out in accordance with the ethical guidelines for medical and health research involving human subjects (http://www.mhlw.go.jp/file/06-Seisakujouhou-10600000-Daijinkanboukouseikagakuka/0000080278.pdf).

We performed BV screening tests during the EGP and MGP at our hospital from July 2004 to April 2014. Patients who subsequently delivered at our hospital were subjects of this study. Patients who were referred from other hospitals from GW 20 onwards, patients with multiple pregnancies, and patients with polyhydramnios due to fetal malformations were excluded. Vaginal or cesarean section deliveries <GW 37 due to onset of labor, intrauterine infection, or cervical dilatation were counted as preterm delivery events. Deliveries due to non-reassuring fetal status, gestational hypertension, placental abruption, maternal complications, or intrauterine fetal death were counted as termination of pregnancy in advance.

We have also screened chlamydia trachomatis infection at the cervical canal during the EGP. Affected women were treated by oral administration of 2 grams of Azithromycin hydrate.

### BV screening and treatment

The Nugent score (NS)^7^ was used to perform BV screening. Vaginal discharge was Gram-stained and scored based on the elimination of *Lactobacillus* and increases in *Gardnerella* and *Mobiluncus* species. Scores of 0–3 were diagnosed as normal (N), scores of 4–6 were diagnosed as Intermediate (I), and scores of 7–10 indicated BV.

BV patients were treated by administering metronidazole vaginal tablets for 6 days (250 mg × 6 days). These were inserted by the physician at the outpatient department on the first day, then inserted by the patients themselves on the remaining 5 days.

### Group 1: Observation group

Group 1 was comprised of patients who underwent prenatal check-up at our hospital from July 2004 to December 2008. In principle, vaginal secretion was taken to examine the presence of group B streptococcus (GBS) during the EGP (<GW 20 weeks) and MGP (GW 20 to GW 33 weeks) for the purpose of preventing neonatal GBS infection by administrating antibiotic to parturient women having vaginal GBS. We performed BV screening tests using the samples used for the GBS tests with the approval of all the patients. Because evidences to treat BV were not enough during this period, we did not treat the BV patients just by the Nugent score. If apparent or symptomatic vaginitis was observed, then patients were treated and followed up over time.

### Group 2: Treatment group

Group 2 was comprised of patients who underwent prenatal check-up at our hospital from July 2010 to April 2014. Because the 2007 Cochrane review stated that treating BV with antibiotics up to GW 20 prevents preterm delivery^6^, we changed our routine practice from that adopted in Group 1. In principle, we performed BV screening tests during the EGP, and as a rule, BV was treated if it was detected, then the patients were retested during the MGP.

### Statistical methods

The preterm delivery rate was tested using the log-rank test, and other analyses were performed using the methods stated in the Results. In order to avoid a multiple testing, when we compared three or more cumulative delivery rate curves at once, we got a single P value testing the null hypothesis that all the samples come from populations with identical preterm delivery rate, and that all differences are due to chance. Correlation between NS in the EGP and NS in the MGP in Group 1 was analyzed by Spearman’s correlation test. The ratios of BV, I, and N subgroups were compared between EGP and MGP using chi-squared test. Paired analysis comparing NS between EGP and MGP was performed by Wilcoxon matched-pairs signed rank test. Comparison of BV cases at EGP for vaginal bacteria status at MGP between Group 1 and Group 2 was performed by Fisher’s exact test. All the statistical analyses were performed using GraphPad Prism Version 6.0 software, and a p value of <0.05 was considered significant.

## Results

### Patient characteristics

**Figure 1:**
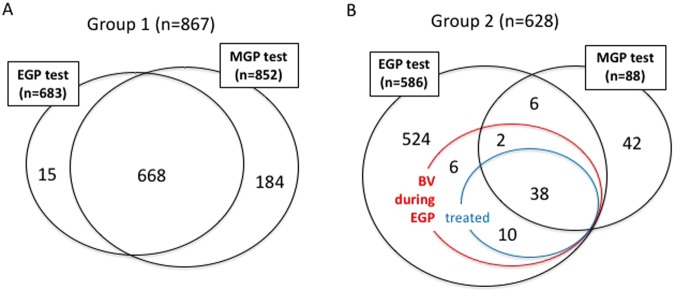
Venn diagram to classify the group 1 and group 2 cases. (**A**) In principle, group1 cases underwent BV tests during both the EGP and the MGP. EGP tests, MGP tests, and both tests were performed for 683, 852, and 668 women, respectively. (**B**) In principle, group 2 cases underwent BV tests during the EGP, then BV cases were treated, followed by BV tests during the MGP. EGP tests and MGP tests were performed for 586 and 88 women, respectively. 56 women were BV positive during the EGP.

Group 1 comprises of 867 patients tested for BV during the EGP and/or MGP from 2004 to 2008 (Fig. 1A). 668 patients were tested during both the EGP and MGP.

Group 2 comprises of 628 patients tested for BV during the EGP and/or MGP from 2010 to 2014 (Fig. 1B). 586 patients were tested during the EGP, 48 out of 56 BV patients were treated with metronidazole. 38 BV patients were treated and retested.

Patient characteristics are shown in Table 1. We did not exclude past cervical conization or chlamydia trachomatis cases because exclusion of these cases did not substantially change the following results (data not shown).

**Table 1:** Patient characteristics. SD; standard deviation. PP; primipara. MP; multipara. HDP; hypertensive disorders in pregnancy. FGR; fetal growth restriction. IUFD; intrauterine fetal death.

	Group 1	Group 2
Total number of cases	867	628
Age at delivery (mean ± SD)	30.4 ± 5.2	32.0 ± 5.1
Obstetric history (PP/MP)	480/387	318/310
Chlamydia trachomatis	26 (3.0%)	7 (1.1%)
Past cervical conization	5 (0.6%)	4 (0.6%)
BV at EGP and/or MGP	50 (5.8%)	60 (9.6%)
Preterm delivery + termination in advance	58 (6.7%)	37 (5.9%)
Preterm delivery	51 (5.9%)	28 (4.5%)
Reason of termination in advance	HDP 3, FGR 1, IUFD 1, placenta previa 1, thinning of surgical scar at uterine muscle 1	HDP 4, abruptio placentae 2, FGR 2, maternal complication 1

### BV test results in group 1

**Figure 2:**
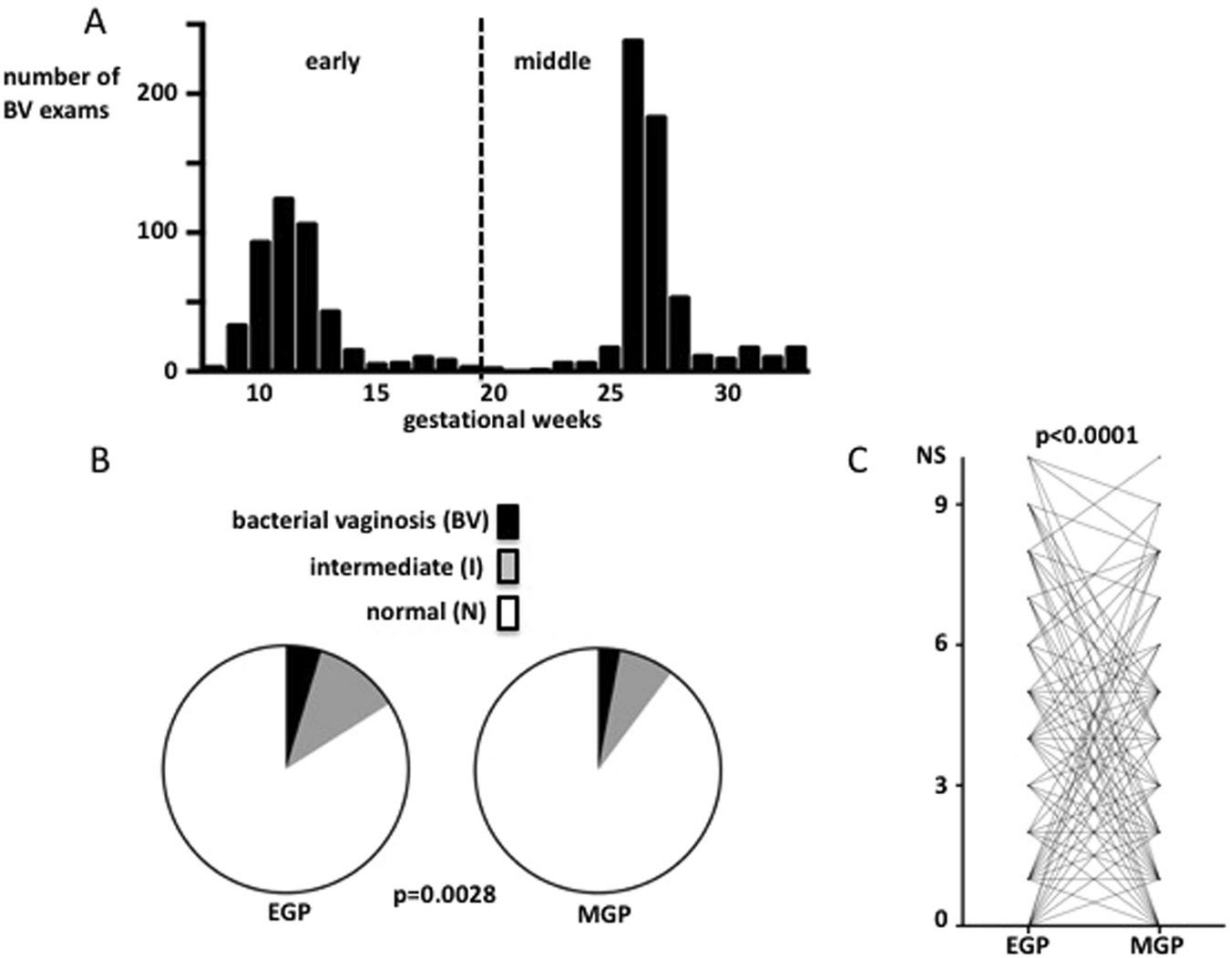
Outline of bacterial vaginosis (BV) exams in group 1. NS 7–10: bacterial vaginosis (BV); NS 4–6: intermediate (I); NS 0–3: normal (N). (**A**) Number of BV exams stratified by gestational week. (**B**) Comparison of ratios of BV, I and N cases between the early gestational period (EGP) and middle gestational period (MGP). (**C**) Change in Nugent score (NS) from EGP to MGP in 668 paired cases.

The average gestational weeks of the EGP and MGP tests were 12.0 and 27.4, respectively (Fig. 2A). Among the 668 patients tested for BV during both the EGP and the MGP, a significant correlation was observed between the NS in the EGP and the NS in the MGP (r = 0.39, p < 0.0001, Spearman’s correlation test). However, there were 32 patients (4.7%) in the BV subgroup, 78 patients (11.4%) in the I subgroup, and 573 patients (83.9%) in the N subgroup during the EGP, and 25 patients (2.9%), 62 patients (7.3%), and 765 patients (89.8%), respectively, during the MGP. These results show that the BV and I subgroups were significantly smaller in the MGP than in the EGP (p = 0.0028, chi-squared test, Fig. 2B). Furthermore, paired analysis of the 668 patients who were tested for BV in both the EGP and MGP showed that the NS was significantly lower in the MGP than the EGP (p < 0.0001, Wilcoxon matched-pairs signed rank test, Fig. 2C).

### Relationship between group 1 BV test results and preterm delivery rate

**Figure 3:**
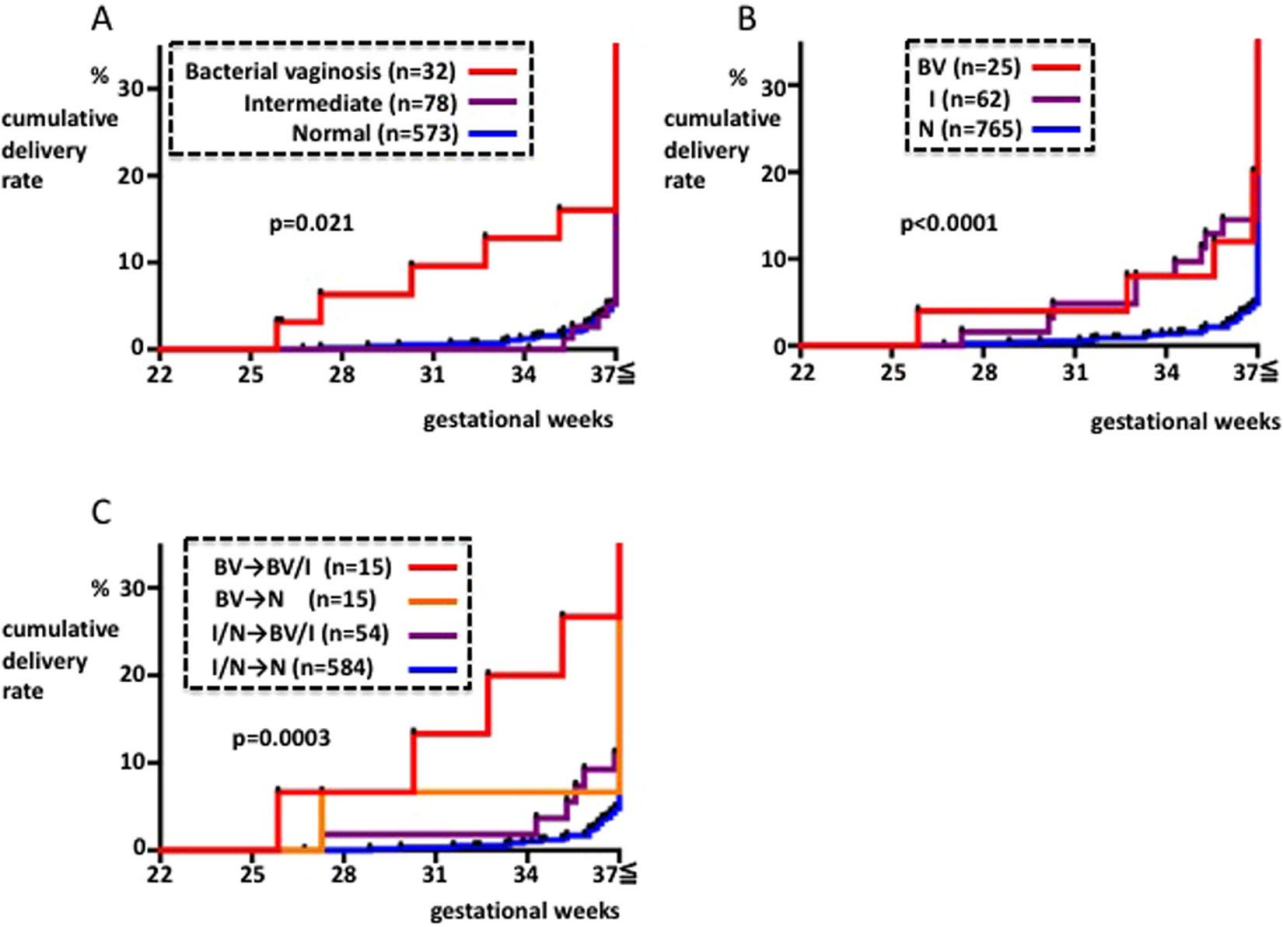
NS and ratio of preterm delivery in group 1. X-axis: gestational period. Y-axis: cumulative ratio of delivery. Cases are divided by (**A**) NS during the EGP (<20 weeks), (**B**) NS during the MGP (20–33 weeks) or C) NS during the EGP and MGP. The arrows indicate changes from the EGP to the MGP.

Patients who were tested for BV during the EGP were divided into three subgroups, BV, I, and N, and then analyzed. There were significant differences between these three subgroups in terms of preterm delivery rate (p = 0.021, Fig. 3A). Cumulative delivery rate curves from the I and N subgroups were similar; the preterm delivery rate was higher in the BV subgroup than in the I and N subgroups. Additionally, using test results from the MGP, the cumulative delivery rate curves from the BV and I subgroups were similar. This revealed a lower preterm delivery rate in the N subgroup than in the BV and I subgroups, and significant differences were observed between the three subgroups (p < 0.0001, Fig. 3B). These results show that patients are at risk of preterm delivery in the BV subgroup during the EGP and in the BV and I subgroups in the MGP.

Next, we created four new subgroups for analysis. These subgroups were the BV → BV/I subgroup, composed of the patients in the EGP BV subgroup who fell into the BV or I subgroup during the MGP; the BV → N subgroup, composed of patients from the EGP BV subgroup who fell into the N subgroup during the MGP; the I/N → BV/I subgroup, composed of patients from the EGP I or N subgroup who fell into the BV or I subgroup during the MGP; and the I/N → N subgroup, composed of patients from the EGP I and N subgroups who fell into the N subgroup during the MGP. Our analysis of the four subgroups showed that there were significant differences in terms of the preterm delivery rate between them (p = 0.0003, Fig. 3C). Among these subgroups, the preterm delivery rate was highest in the BV → BV/I subgroup and lowest in the I/N → N subgroup.

### Relationship between the group 2 BV test results and the preterm delivery rate

**Figure 4:**
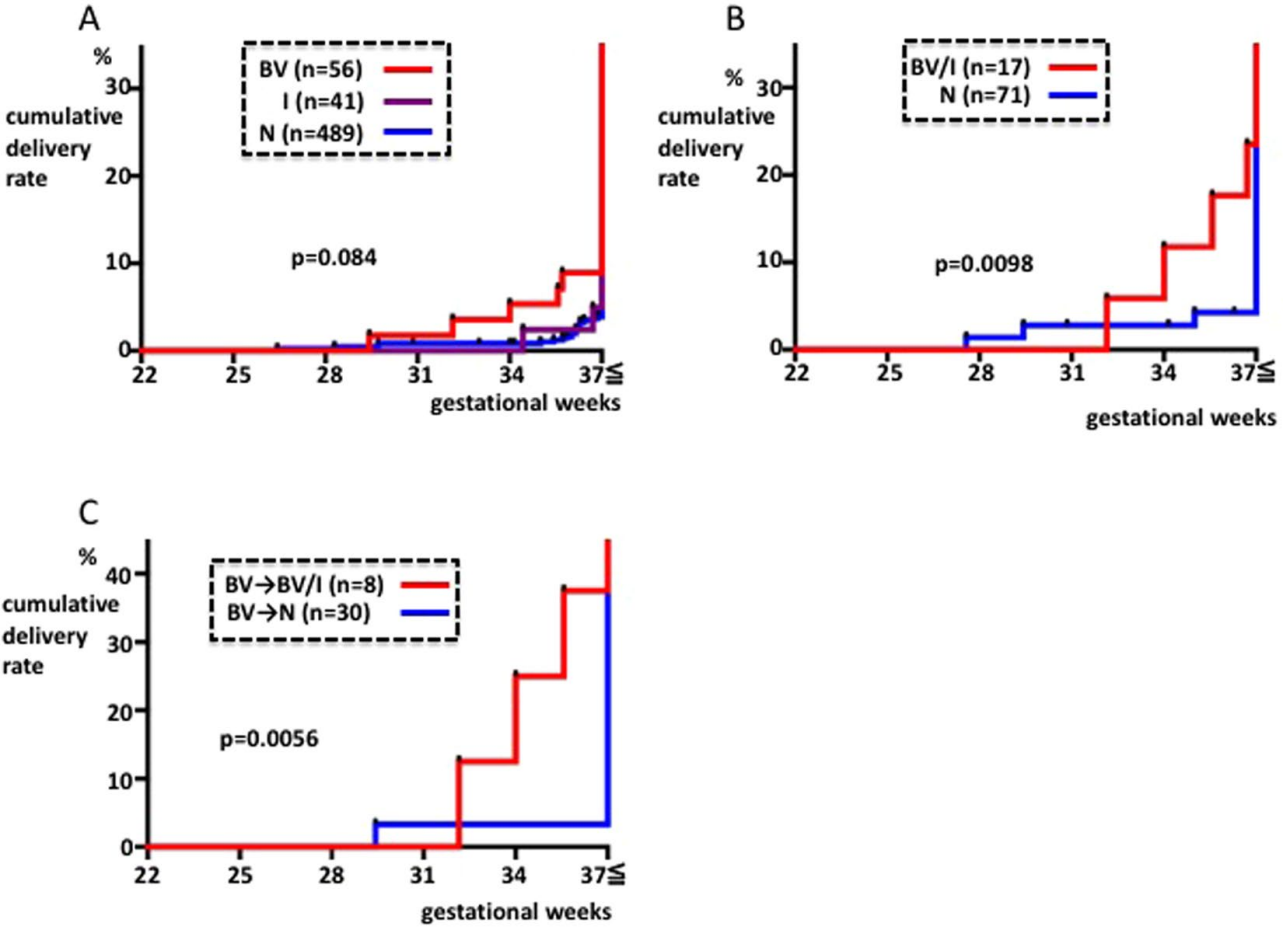
BV status and ratio of preterm delivery in group 2. X-axis: gestational period. Y-axis: cumulative ratio of delivery. (**A**) Comparison of the BV, I and N subgroups during the EGP. (**B**) Comparison of BV/I and N subgroups during the MGP. (**C**) Comparison of the BV/I and N subgroups during the MGP following BV treatment during the EGP.

586 patients were examined during the EGP (average 11.4 weeks) in group 2. Comparing the rate of preterm delivery between these subgroups, we found the cumulative delivery rate curves from the I and N subgroups were similar, as seen in group 1. The preterm delivery rate looked slightly higher in the BV subgroup than in the I or N subgroup, but there were no significant differences observed between the three subgroups (p = 0.084, Fig. 4A). This may be because the preterm delivery rate decreased in response to treatment of the BV subgroup.

88 patients were examined during the MGP (average 25.1 weeks). The test results were BV in six patients (6.8%), I in 11 patients (12.5%), and N in 71 patients (80.7%). Due to the low number of patients, the BV and I subgroups were combined into the BV/I subgroup. When we compared the preterm delivery rates in the BV/I subgroup to those in the N subgroup, the rate was significantly higher in the former (p = 0.0098, Fig. 4B), as seen in group 1.

Figure 4C shows the BV subgroup during the EGP, which comprises of 38 patients who were previously treated with metronidazole vaginal tablets and were tested for BV during the MGP. The preterm delivery rate was compared between the eight patients with a treatment outcome of BV or I and the 30 patients with a treatment outcome of N. It was found to be higher in the BV/I subgroup (p = 0.0056, Fig. 4C).

### Comparison of groups 1 and 2

**Figure 5:**
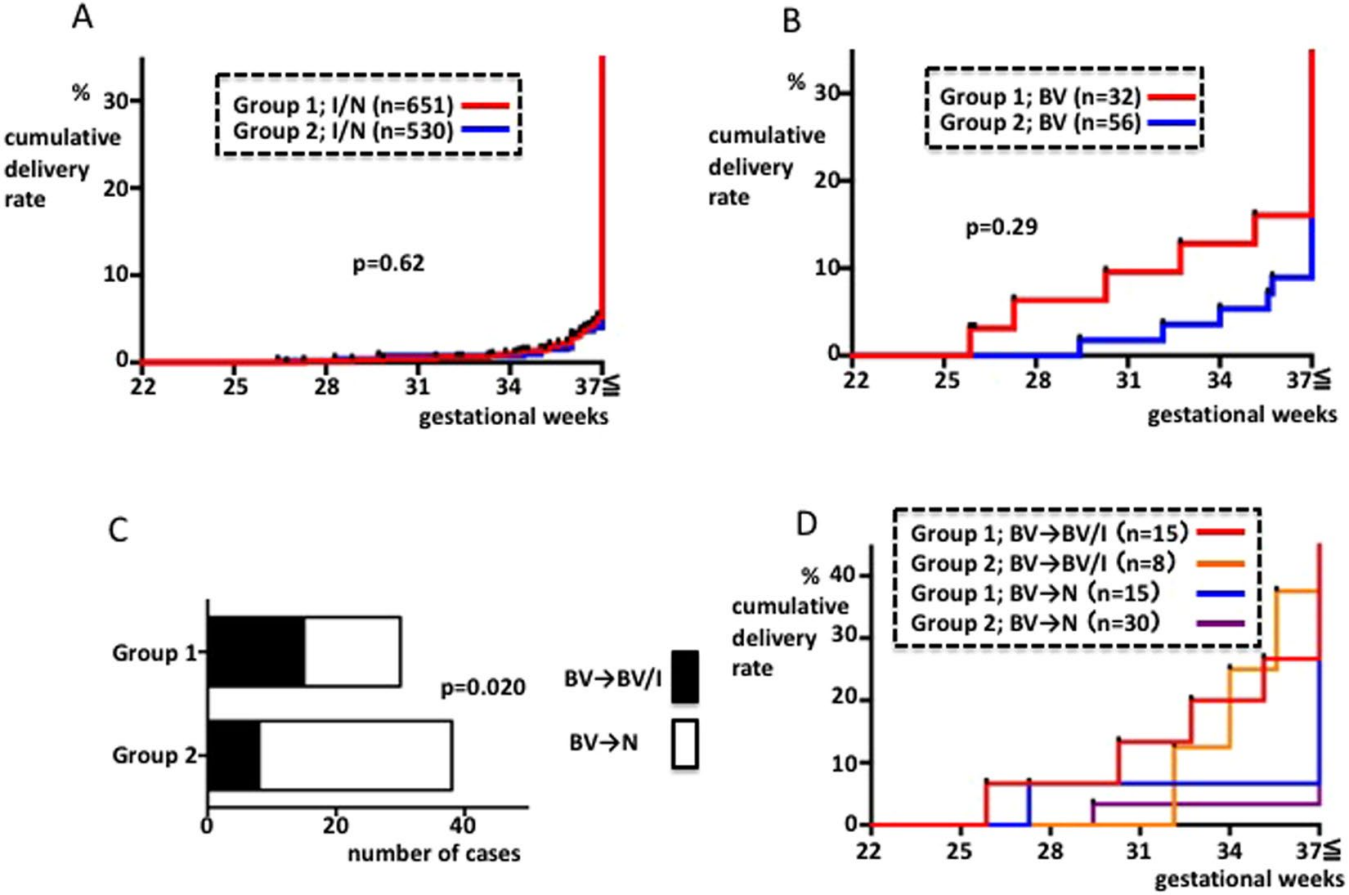
Comparison between group 1 and group 2. (**A**) Ratio of preterm delivery among I/N patients during the EGP. (**B**) Ratio of preterm delivery among BV patients during the EGP. (**B**) Change in vaginal bacteria status of the BV patients from the EGP to the MGP. (**C**) A significant increase in N cases in group 2 compared with group 1 was observed. (**D**) Ratio of preterm delivery among BV patients during the EGP. In (**A**), (**B**), and (**D**), the X-axis shows gestational period. Y-axis: cumulative ratio of delivery.

The cumulative delivery rate curves from the patients of groups 1 and 2 who tested I or N (i.e., I/N) during the EGP were similar and found no significant differences between these groups (p = 0.62, Fig. 5A). We compared patients who tested BV in the EGP and were untreated (group 1) *vs*. treated (group 2) and found that there was a slightly lower preterm delivery rate in group 2, although this was not significant (p = 0.29, Fig. 5B). When we calculated the change in vaginal bacterial status between EGP and MGP, we found that 50% (15/30) of EGP BV patients in (untreated) group 1 tested N in the MGP, compared with 79% (30/38 patients) in (treated) group 2. This result indicated significant improvement in the BV status (p = 0.020, Fisher’s exact test, Fig. 5C). We superimposed the cumulative delivery rate curves from groups 1 and 2, focusing on the patients diagnosed with BV during the EGP and the BV and I/N patients during the MGP (Fig. 5D). We found that if BV was present in the EGP, irrespective of whether treatment was performed, then the preterm delivery rate was based on the test results in the MGP.

## Discussion

In this study, we evaluated the relationship between the natural history of BV during pregnancy and preterm delivery rate in group 1, patients from 2004 to 2008. Furthermore, we compared them with the patients in group 2 from 2010 to 2014 to study the therapeutic effects of metronidazole vaginal tablets. This was a retrospective study, but we were able to analyze the therapeutic effects because we had changed the treatment plan for BV during the relevant period.

In group 1 patients, who were untreated from 2004 to 2008, the vaginal bacteria status spontaneously improved in the MGP compared with the EGP, so the BV subgroup decreased in size (Fig. 2B,C). Until that time, one study had investigated the vaginal bacteria status in 718 pregnant patients and reported that approximately 50% of BV in the EGP had improved and normalized^10^. These results are consistent with our current results. The reason for this improvement could be the increased quantity of glycogen in the vaginal epithelium, which increases Lactobacillus count^11^, although the precise mechanism is unclear and remains a challenge for future research.

There have been several reports about the increased risk of preterm delivery when patients test positive for BV and consensus has been reached^12–21^. However, no consensus has been reached in terms of the I subgroup, as there are reports that state that there is a risk of preterm delivery^15,17,18^ as well as at least one report stating that there is not^20^. This may be because the tests were not performed at the same gestational period in every report and because the spontaneous remission of BV was not recognized. Our results show that there was an increased preterm delivery rate in the BV subgroup during the EGP and in the BV and I subgroups in the MGP (Fig. 3). We found these results because we were able to observe the natural course following the BV tests during both the EGP and the MGP. To the best of our knowledge, this is the first study of its kind. We believe that it is appropriate to evaluate the risk of preterm delivery using vaginal culture during the EGP and MGP. To establish these guiding principles for the future, we will need to perform larger-scale analyses using the same methods and the reproducibility of our results will need to be confirmed.

Group 2, comprising patients who were followed from 2010 to 2014, were tested for BV during the EGP as a rule and were treated with metronidazole if they fell into the BV subgroup. A direct comparison between the BV patients in groups 1 and 2 during the EGP showed that the differences in the preterm delivery rates were not statistically significant (Fig. 5B). Meanwhile, when we examined vaginal flora, the N patients increased significantly in group 2 compared with group 1 (Fig. 5C). Several reports have investigated the presence of effects that prevent preterm delivery after metronidazole administration in BV patients in the EGP, although most of these involved oral administration. Some reports state that there are effects that prevent preterm delivery^20–22^, although numerous recent, large-scale, randomized, comparative studies have found no effects^23–26^. Meanwhile, numerous studies have noted therapeutic effects after oral or vaginal administration of metronidazole, based on vaginal flora findings^27–32^. During our study, we administered metronidazole vaginally, so there were differences in the administration route from the previous studies. However, we believe our results are consistent with those from previous reports, as we did not observe effects that prevented preterm delivery but did observe an improvement in the vaginal findings.

The reason that the results of the current study do not show preventive effects on preterm delivery, despite treatment of the BV patients in the EGP, may be because there were patients who did not fall under the N subgroup in the MGP and because those patients were at increased risk of preterm delivery (Fig. 4C,D). Metronidazole treatment usually cures BV because lactobacilli are resistant to metronidazole, whereas most of BV-causing bacteria are sensitive to metronidazole^31^. However, in this study, insufficient patient compliance or a recurrence, which can be caused by sexual intercourse^32–34^, after an initially successful treatment at EGP, may be the reason of the apparently treatment-resistant cases. Additionally, treatment failure of BV can be caused by metronidazole-resistant Gardnerella vaginalis^35,36^ or lack of lactobacilli^37^, and therefore, new forms of treatment may decrease the size of this treatment-resistant subgroup. For example, Giunta *et al*. showed that the preterm delivery rate improves after administration of lactoferrin^38^. If these methods are combined, then the ratio that enters the N subgroup during the MGP may approach 100% and may be associated with decreased preterm delivery rates.

One of the limitations of this study was that it was performed using historical controls, although the results suggest that there were no major differences between groups, except for the presence or absence of metronidazole vaginal tablet administration in the group 1 and 2 BV patients. Patients who fell into the I or N subgroup during the EGP, i.e., patients who were not treated with metronidazole, showed an unchanged cumulative delivery ratio < GW 37 when compared with groups 1 and 2 on the whole. The preterm delivery rate was low during all periods (Fig. 5A). Another limitation of this study was the small sample size. Non-significant differences observed in this study (i.e. Figs 4A and 5B) could be attributed to the small sample size. Given the low frequency of BV cases, randomized clinical trials involving large number of pregnant women would be required to develop a standardized treatment strategy for BV.

In conclusion, our current results show that BV often improved spontaneously during pregnancy. In addition, the BV subgroup in the EGP and the BV or I subgroup in the MGP were at risk of preterm delivery. Vaginal bacterial findings improved when patients in the EGP BV subgroup were treated with metronidazole vaginal tablets for six days. However, due to the presence of some treatment-resistant patients, we did not observe any significant preventive effects on preterm delivery. The results of this study suggest a method for using intravaginal bacterial findings during pregnancy as a biomarker to evaluate the risk of preterm delivery. Further large-scale randomized trials are necessary.
